# Zein/MCM-41 Nanocomposite Film Incorporated with Cinnamon Essential Oil Loaded by Modified Supercritical CO_2_ Impregnation for Long-Term Antibacterial Packaging

**DOI:** 10.3390/pharmaceutics12020169

**Published:** 2020-02-18

**Authors:** Xiaojing Liu, Jingfu Jia, Shulei Duan, Xue Zhou, Anya Xiang, Ziling Lian, Fahuan Ge

**Affiliations:** 1 School of Traditional Chinese Medicine, Guangdong Pharmaceutical University, Guangzhou 510006, China; LXJ_18826238139@163.com (X.L.); 17854223645@163.com (S.D.); 2 School of Pharmaceutical Sciences, Sun Yat-sen University, Guangzhou 510006, China; zhouxue9@mail.sysu.edu.cn (X.Z.); xiangany@mail2.sysu.edu.cn (A.X.); lianzling@mail2.sysu.edu.cn (Z.L.)

**Keywords:** film nanocomposite, essential oil, supercritical CO_2_, long-term package

## Abstract

Antimicrobial medicine and food packages based on bio-based film containing essential oils have attracted great attention worldwide. However, the controlled release of essential oils from these film nanocomposites is still a big challenge. In this study, a long-term antibacterial film nanocomposite composed of zein film and cinnamon essential oil (CEO) loaded MCM-41 silica nanoparticles was prepared. The CEO was loaded into MCM-41 particles via modified supercritical impregnation efficiently with a high drug load (>40 *wt%*). The morphologies of the prepared nanoparticles and film nanocomposite were characterized by a scanning electron microscope. The release behaviors of CEO under different temperatures, high humidity, continuous illumination and in phosphate buffer solution (PBS) solution were investigated. The results showed that the film nanocomposite had an outstanding release-control effect. The addition of MCM-41 nanoparticles also improved the mechanical properties of zein films. The antibacterial effect of CEO was significantly prolonged by the film nanocomposite; indicating the CEO film nanocomposite fabricated via modified supercritical CO_2_ impregnation was a potential long-term antibacterial medicine or food package material.

## 1. Introduction

With increasing concerns about the environment, ecology and safety in the last decade, biodegradable medicine and food packaging materials have gained more and more attention all over the world [1]. These film materials normally originated from regenerative resources such as proteins, lipids, cellulose, polysaccharides, lactic acid, and so on [2,3,4]. Furthermore, for the purpose of extending the shelf-life or avoiding microbial contamination, active packaging materials generated via the incorporation of bio-based films and antimicrobial components such as essential oils, were developed [5,6]. Several studies have shown that adding essential oils into polymeric films significantly enhanced the antibacterial properties of the package [7,8,9]. However, the components in essential oils generally have some inherent drawbacks such as high volatility and decomposing tendency. Therefore, the essential oil was lost rather fast from the polymeric films, and this short lifespan of active components could hardly meet the needs of long-term activity for package application [10]. Therefore, the development of controlled-release films was demanded.

Encapsulating essential oil into nanoparticles and incorporating these nanoparticles with bio-based film to fabricate film nanocomposite is an effective strategy to control the release of essential oils. It has been reported that chitosan or silicate nanoparticles could help essential oils release in a slow but sustained way, as well as improve their stability and long-term antimicrobial effect [11,12,13]. However, encapsulating or loading essential oils into nanoparticles is still a big challenge. In most of the process that was reported, large amounts of organic solvents or surfactants were used, and the drug loads were unsatisfactory (<10 *wt*%) [11,14,15]. Thus, nanoparticles in some film nanocomposite containing essential oil only played the role of improving the film mechanical properties, but not as a drug carrier [16].

Supercritical CO_2_ technology may be an alternative to loading essential oils into mesoporous nanoparticles with high efficiency [17,18]. Many natural active components including essential oils have considerable solubility in supercritical CO_2_, making the supercritical CO_2_ a good vehicle for transporting essential oil. Furthermore, supercritical CO_2_ possesses strong permeability benefit from its gas-like viscosity, and its solvation stability will be deprived after the phase transformation of supercritical to gaseous state, which can be achieved readily by operation adjustment [19]. Therefore, supercritical CO_2_ can be a good vehicle for essential oil to transport into nanoparticles and make no solvent residue after drug loading. In our previous study, essential oil was loaded into mesoporous silica nanoparticles with a high drug load (>35 *wt*%) using a modified supercritical CO_2_ impregnation method [20].

Cinnamon essential oil (CEO) has been reported to have good antimicrobial effects [21,22], and its leading component is cinnamaldehyde. The aim of this study was to develop a biodegradable long-term antibacterial film nanocomposite for medicine or food packaging, based on the combination of zein film and CEO loaded silica nanoparticles. To the best of our knowledge, this type of film nanocomposite having a long-term antibacterial effect for medicine or food package has not been reported. Supercritical CO_2_ technology was used to load CEO into MCM-41 silicate nanoparticles. Furthermore, the mechanical properties and antibacterial effect of the prepared film nanocomposite were evaluated.

## 2. Materials and Methods

### 2.1. Materials

Zein powders extracted from corn germ were delivered by Sinopharm Chemical Reagent Co., Ltd. (Shanghai, China). CEO (Cinnamaldehyde content >95%) of pharmaceutical grade with quality inspection meeting the Chinese Pharmacopoeia standards was supplied by Jiangxi Anbang Pharmacy Co., Ltd. (Ji’an, China). Tetraethyl orthosilicate (TEOS, >99%), cetyltrimethyl ammonium bromide (CTAB, >99%), diethanol amine (DEA, >99%), glycerol (>99.7%, GC), and standard substance of cinnamyl aldehyde (>99.5%, GC) were purchased from Aladdin (Shanghai, China). Carbon dioxide (99.99%) was obtained from Guangzhou Gas Factory Co., Ltd. (Guangzhou, China). Tryptone agar and yeast extract were from Coolaber Science & Technology Co. Ltd. (Beijing, China). Stock culture of *Staphylococcus aureus* (ATCC 27217) was obtained from Solarbio Science & Technology Co., Ltd. (Beijing, China). Deionized water was produced by a Milli-Q system and acetonitrile was chromatographically pure. Other reagents were analytical grade and used directly.

### 2.2. Preparation of CEO Loaded Silica Nanoparticles (CEO@MCM-41)

The mesoporous silica nanoparticles MCM-41 were prepared using the method described previously [23]. Typically, 0.4 mol CTAB and 0.062 mol DEA was mixed in 165 mL water and stirred at 350 r/min under 95 °C, then 1 mol TEOS was dropwise added within 45 min. After reacting for another 1 h, the white emulsion was centrifuged and washed with water and methanol several times. Finally, the obtained particles were soaked in ethanol/HCl (8/1, *v*/*v*) at 60 °C for 24 h to remove the surfactant template, resulting in CTAB free MCM-41.

CEO was loaded into MCM-41 by modified supercritical CO_2_ impregnation and the equipment was described in a previous study [20]. In this step, 200 mg MCM-41 particles were firstly put in a stainless basket with an air-permeable bottom and sealed in a high-pressure drug loading kettle, where a magnetic stirring apparatus was loaded at the kettle base. Next, 2 mL CEO was injected into the kettle to make the MCM-41 particles soaked for 15 min. Afterwards, CO_2_ was delivered into the kettle continuously until the pressure rose to 15 MPa, and the kettle was heated to 40 °C at the same time. This supercritical state was kept for 1 h to load CEO into MCM-41 particles, then the kettle temperature was cooled below 20 °C followed by depressurization. The procedure of “CEO injection, soak, CEO loading under supercritical state and depressurization” was cycled five times to make an almost saturated drug load. The final obtained CEO@MCM-41 particles were stored at 4 °C.

### 2.3. Film Nanocomposite Fabrication

Zein-based filmed nanocomposite was prepared by a casting method. Firstly, 2 g zein and 0.5 g glycerol were dissolved in 97% ethanol solution (10 mL) at 70 °C and stirred for 1 h at 300 rpm through magnetic stirring. Then, a certain amount of CEO@MCM-41 particles were added into the zein solution, and stirred for another 30 min. Finally, the solution was casted in Teflon molds and dried at 40 °C for 8 h. Thus, the CEO@MCM-41/zein film nanocomposite was obtained and peeled off for later use.

By the same methods, zein-based film containing blank MCM-41 (blank film nanocomposite) or CEO alone (CEO/zein film) were prepared, where the amounts of MCM-41 and CEO used were equal to those in the film nanocomposite.

### 2.4. MCM-41 Particles Characterization

Brunner−Emmet−Teller (BET) and Barrett-Joyner-Halenda (BJH) measurements were carried out to examine the internal pore structures of MCM-41 and nitrogen-adsorption-desorption isotherms were plotted using a specific surface meter (JW-BK200C, JWGB Sci. & Tech. Co., Ltd., Beijing, China). The morphology of MCM-41 particles were observed by a high-resolution scanning electron microscope (SEM, Gemini500, Zeiss/Bruker, Karlsruhe, Germany).

### 2.5. Film Nanocomposite Characterization

#### 2.5.1. Morphology

The morphology of the film nanocomposite was observed by a scanning electron microscope (SEM, JSM-6330F, JEOL Ltd., Tokyo, Japan) of both surface and cross section. The samples were attached on an electrically conductive adhesive stuck to an aluminum stub and then coated with platinum using a sputter coater (1.2 kW, E-1045, Hitachi, Tokyo, Japan) for 120 s.

#### 2.5.2. Film Thickness and Mechanical Properties

The film thickness was determined using a professional digital display thickness gauge (EXPLOIT, Jinhua, China), which had a sensitivity of 0.001 mm. Different locations (>6) of the film were picked randomly to be measured, and an average value was considered the final thickness value.

For tensile strength (TS) and elongation at break (EAB%) determination, the film was firstly stored under 50% humidity for 60 h before being cut into 5 × 25 mm rectangular strips. The strips were then determined by a fastener tension tester (QJ212, Qingji, Shanghai, China). Parallel determinations of ten times for each sample were carried out.

### 2.6. Contents and Release Behavior of CEO

As only one effective peak referring to cinnamaldehyde was displayed in the chromatogram (see Figure S1 in the Supporting Materials), the amount of CEO was calculated based on the determination of cinnamaldehyde (96.8% in the CEO used) using a high-performance liquid chromatography system (HPLC, UltiMate 300, Thermo Fisher Scientific Ltd., Waltham, MA, USA). During the determination, a Sharpsil-HC18 chromatography column (250 L × 4.6 mm I.D., S-5 μm 100A) was used, with a gradient acetonitrile/water mobile phase, in which the acetonitrile ratio increased gradually from 35% to 38% within 0–10 min, and continued to rise to 50% within 10–15 min. The samples were detected by UV at 290 nm, the temperature was 30 °C, and the flow rate was 1 mL/min.

The release behaviors of CEO from CEO/zein film, CEO@MCM-41 and film nanocomposite under high temperature (40, 60 and 80 °C, humidity of 40%), high humidity (80%, 25 °C), continuous illumination, and in phosphate buffer solution (PBS, pH 7.0) were investigated. For temperature and humidity tests, several precisely weighed samples were put into a constant temperature and humidity chamber (LHS-80HC-I, Yiheng, Shanghai, China), and one of them was taken out at preset time intervals. The fetched samples were immediately dissolved in 90% ethanol solutions for HPLC determination.

For the illumination test, the samples were put in a clean fuming cupboard with the light open, but the window of the cupboard was shielded by a thick black curtain from external light. The sampling and determination methods were the same as the temperature and humidity tests.

For the release test in PBS, 50 mg CEO@MCM-41 nanoparticles or 100 × 100 mm film samples (precisely weighed) were directly put into 120 mL PBS solution with 180 rpm magnetic stirring. At each time interval, 1 mL solution was withdrawn from the same water level for HPCL determination, and 1 mL PBS solution was replenished immediately.

### 2.7. Antibacterial Activity

The antibacterial activity of the CEO/zein film (1.0 *wt*%) and CEO film nanocomposite (1.0% CEO@MCM-41 nanoparticles) were tested against *Staphylococcus aureus* (*S. aureus*, LA9190, Solarbio, Beijing, China) using the plate diffusion method, as well as the CEO solution (2 mg/mL in ethanol) and blank film nanocomposite for the control. 25 mL culture medium (Macklin, Shanghai, China) was firstly put in a 100 mm diameter plate, and 0.4 mL inoculum containing 4 × 10^4^ CFU/mL bacteria was spread after the medium cooled. Then an oxford cup was set in the center, and 250 μL CEO solution or 340 mg film sample was put together with 1 mL ethanol into the oxford cup carefully. Finally, the plates were incubated at 36 °C for 20 h before observing the inhibitory zone surrounding the oxford cup.

## 3. Results and Discussion

### 3.1. Morphology and Structures of MCM-41 and CEO@MCM-41

**Figure 1:**
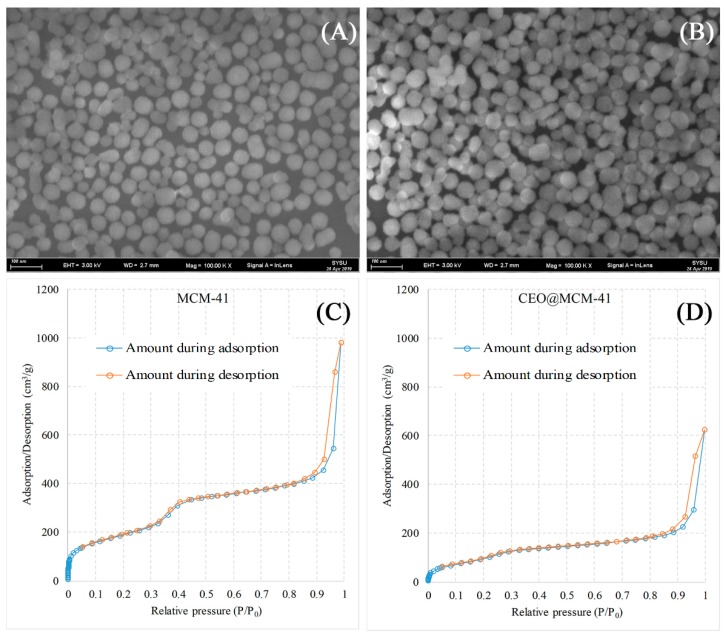
Scanning electron microscope images of (**A**) MCM-41 and (**B**) CEO@MCM-41 particles, and nitrogen-adsorption–desorption isotherms of (**C**) MCM-41 and (**D**) CEO@MCM-41 particles.

The morphology of the prepared silica MCM-41 and CEO@MCM-41 particles were observed by SEM, and the images are shown in Figure 1A,B, separately. Both powders had spherical particles with uniform diameters around 50 nm, indicating that loading CEO into MCM-41 by supercritical impregnation brought no change to the particle morphology. The nitrogen-adsorption-desorption isotherms of MCM-41 displayed in Figure 1C demonstrated that uniform mesoporous channels were formed in the particles, as well as CEO@MCM-41 in Figure 1D. Furthermore, it could be seen that the adsorption/desorption volume of CEO@MCM-41was decreased compared to that of MCM-41, indicating that CEO was loaded into the inner pore channels. The BET and BJH determination results including specific surface area, pore volume and pore size are listed in Table 1. It can be seen that MCM-41 had a high BET specific surface area value of 596.36 m^2^/g, and the BJH adsorption and desorption values were close. However, BET surface area decreased to 264.47 m^2^/g for CEO@MCM-41, indicating that CEO was successfully loaded into the mesoporous channels, which was also supported by the obvious differences between the BJH adsorption and desorption values.

### 3.2. Drug Load of CEO in CEO@MCM-41

**Figure 2:**
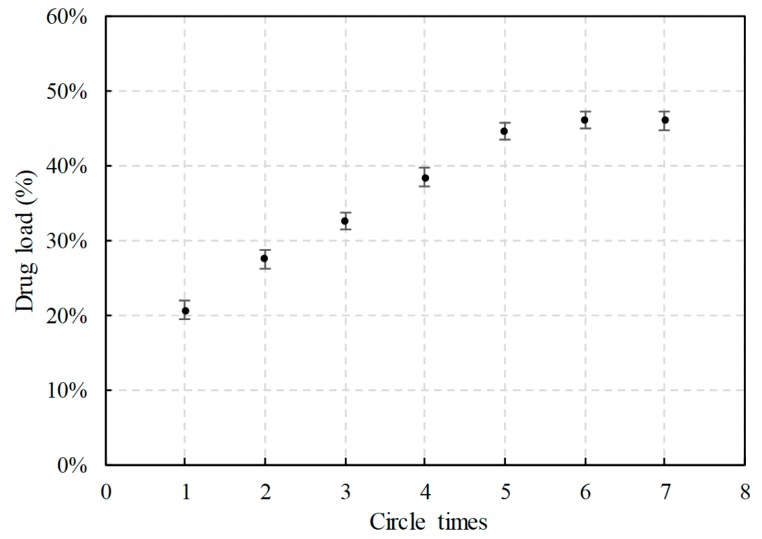
Drug load of CEO@MCM-41 prepared under different circle times by modified supercritical impregnation.

CEO was loaded into MCM-41 via modified supercritical impregnation and the circle time was a crucial factor for the drug load of CEO@MCM-41. In this study, with the circle time increased, the drug load increased rapidly to 44.5 ± 0.6% at the first stage (before five times, Figure 2). However, the growth stopped when the circle time went over five, indicating a saturated drug load was achieved. Multiple circle operations were necessary for getting the maximum drug load and the reason has been discussed in our early study [20]. Simply, partial CEO loaded in the MCM-41 particles during the previous circle could hardly dissolve in supercritical CO_2_ again (or the dissolution rate decreased seriously) in the following circle, due to the strong absorption caused by the silanol groups of MCM-41.

### 3.3. Morphology of the Film Nanocomposite

**Figure 3:**
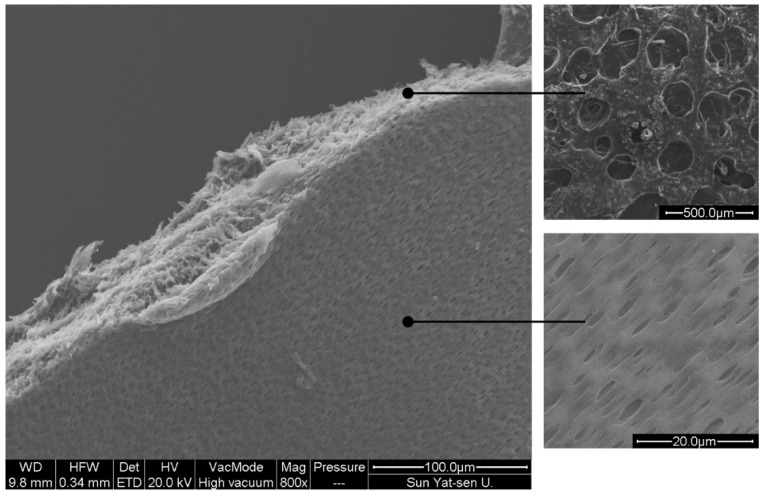
Scanning electron microscope images of the crack edge for the film nanocomposite.

The morphology of the zein-based film containing CEO@MCM-41 nanoparticles, that is film nanocomposite, is shown in Figure 3. It can be seen that lots of pore channels existed in the film nanocomposite, enabling the CEO@MCM-41 nanoparticles to become well dispersed, which impacted the uniformity of film quality such as the mechanical properties and the local release behavior of the loaded CEO. These channels could also help the CEO loaded in the nanoparticles release out from the film. It was interesting to note, that the pore size on the film surface was much smaller than the size of the inner channel, which might be able to slow the CEO release to some extent.

### 3.4. Physical and Mechanical Properties of Zein-Based Films

The physical and mechanical properties of the prepared film nanocomposite is very important for its use in medicine or food packaging, and the film thickness should be considered at first. Shown in Table 2, compared to the blank zein film, adding MCM-41 particles made a thicker film nanocomposite, and the thickness grew slowly with MCM-41 content increase. In comparison, the thickness had no significant change with the addition of 1% CEO. The thickness turned higher when MCM-41 existed, probably attributed to the nanoparticle movement that caused an amplified cavity in the film.

**Figure 4:**
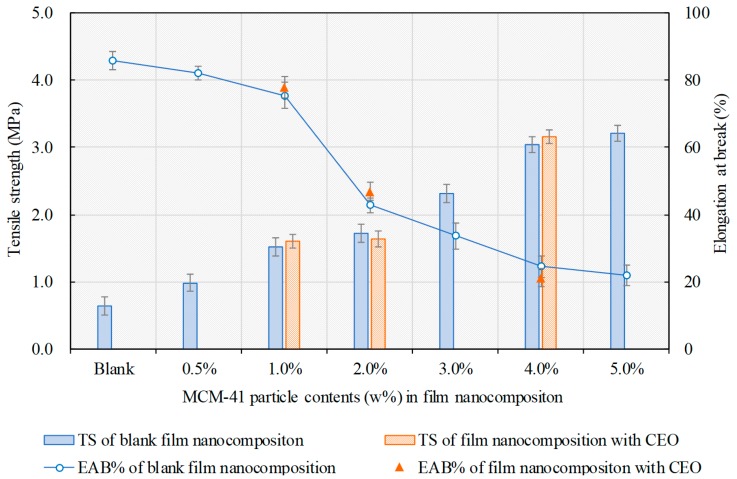
Tensile strength (TS, bars) and elongation at break (EAB%, dots and line) of the film nanocomposite with different MCM-41 particle contents.

Tensile strength (TS) and elongation at break (EAB%) are also crucial parameters for the package-use film nanocomposite. Figure 4 represents TS and EAB% of film nanocomposite with different MCM-41 contents. The TS of the film nanocomposite could be seen without the CEO in MCM-41, which increased gradually with the MCM-41 content from 0.6 ± 0.1 MPa (blank zein film) to 3.2 ± 0.1 MPa (5% MCM-41), while EAB% decreased from 85.9 ± 2.7% to 22.0 ± 3.1%, where a sharp decrease occurred when the MCM-41 particle content increased from 1.0% to 2.0%. The TS reinforced the film nanocomposite by MCM-41 addition, which may have been because of the netted internal hydrogen bonds formed between the nanoparticles and the zein matrix, which enabled the MCM-41 particles to share the drawing force. Similar results were also reported in other studies on film nanocomposite of different types [16]. Meanwhile, although adding lipid substances might modify the mechanical properties of protein-based films via crosslinking effect [24,25], the TS and EAB% had no significant change for the film nanocomposite with or without CEO in this study, as the essential oil was loaded inside the nanoparticles. According to the results, 1.0 *wt*% of MCM-41 nanoparticles in zein film was selected for the following studies.

### 3.5. Release Behavior of CEO from the Film Nanocomposite

The stability and shelf life of CEO in the film nanocomposite was crucial for the duration of the antibacterial effect. In this study, the CEO losses from the CEO/zein film, CEO@MCM-41 and film nanocomposite over time were investigated under different conditions including high temperatures, continuous illumination, high humidity and in PBS solutions. Under all conditions, no newly generated component except for cinnamaldehyde was detected by HPCL, indicating the loss of CEO was mostly caused by the release of cinnamaldehyde or its escape after decomposition.

**Figure 5:**
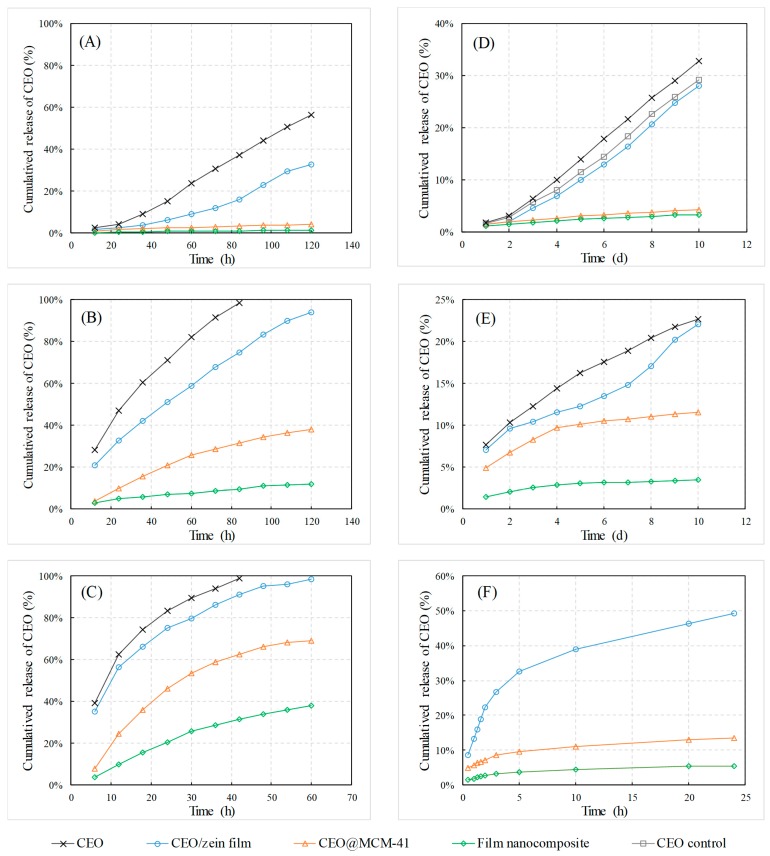
Release behavior of CEO from CEO/zein film, CEO@MCM-41 and film nanocomposite at different conditions. Including: at a high temperature of (**A**) 40 °C, (**B**) 60 °C and (**C**) 80 °C, (**D**) under continuous illumination at room temperature (the control CEO sample without illumination), (**E**) under high humidity of 80% and room temperature, and (**F**) in neutral PBS solution.

Shown in Figure 5A–C, the release rates of CEO exposed to air, as well as in all three carries were accelerated with the temperature increase. The CEO released very fast when exposed to air. For example, it released 37.2% at 40 °C (Figure 5A) and was nearly exhausted at 60 °C (Figure 5B) after a 84 h test. However, the accumulated release amounts of CEO from the CEO/zein film after 84 h at 40 °C and 60 °C dropped to 16.0% (Figure 5A) and 74.56% (Figure 5B), while full release at 80 °C needed 60 h (Figure 5C). Furthermore, the CEO release rate dropped drastically in the order of CEO/zein film, CEO@MCM-41 and film nanocomposite. After 120 h temperature acceleration tests at 40 °C, CEO released only 4.0% and 1.2% from CEO@MCM-41 and film nanocomposite (Figure 5A), respectively. Their accumulated release amounts at 80 °C after 60 h also decreased to 69.1% and 38.2%, compared to CEO/zein film. It could be concluded that both the matrix structure of the zein film and the mesoporous structure helped prolong the life-span of CEO, probably attributed to the hydrogen-bond interaction of CEO molecules provided by the silica hydroxyl inside the MCM-41 particles and the polar groups in the protein matrix.

According to the comparison of CEO release behavior with or without illumination (Figure 5D), it could be seen that illumination was able to accelerate the loss of CEO, which might be attributed to the intensified decomposition and escaping of cinnamaldehyde under light. The release rate from CEO/zein film had a slight decrease. After a 10-day test, CEO released 28.1% from the CEO/zein film, while 32.8% CEO exposed to the air was lost. It was quite different for CEO@MCM-41 and film nanocomposite that CEO gained very low release rate, as the cumulative release amounts of CEO after 10 days were only 4.2% and 3.3%, separately. This was because unlike the transparent zein film, the wall of MCM-41 nanoparticles could prevent CEO molecules from contacting the light.

High humidity could also affect the release behavior of CEO (Figure 5E). After a 10-day test, the cumulative release amounts of CEO exposed to air was 22.7%, which decreased to 22.1% from CEO/zein, 11.6% from CEO@MCM-41, and only 3.5% from film/nanocomposite, respectively. The interesting thing was the released amount from CEO/zein film was less than that of CEO exposed at the beginning, but gradually rose close to the latter. This result indicated that the zein film would loose its protective effect for CEO when it was wetted, because the internal hydrogen bonds were weakened with a water increase. The water-isolation effect of MCM-41 nanoparticles was stronger than that of zein film, since it was more difficult for water to permeate into the nanosized mesoporous channel in the particle. However, high humidity had little effect under the combined use of a zein film and MCM-41, because the matrix structure of the zein film had strong absorption to the permeated water molecules, making the channels of MCM-41 dry.

Compared to the release behaviors in air, CEO released much faster in a PBS solution of pH 7.0 (Figure 5F). CEO/zein film had an initial burst release (22.3%) in the first 2 h, and then released gradually to 49.3% after 24 h. The release rate dropped significantly for CEO@MCM-41 and the film nanocomposite, and the cumulative released amounts were 13.6% and 5.5% at 24 h, respectively. The results demonstrated that the film nanocomposite had an outstanding control-release effect for CEO even in an aqueous solution. This unique property made the CEO film nanocomposite a potential long-acting antibacterial package material for medicine or food.

### 3.6. Antibacterial Properties

**Figure 6:**
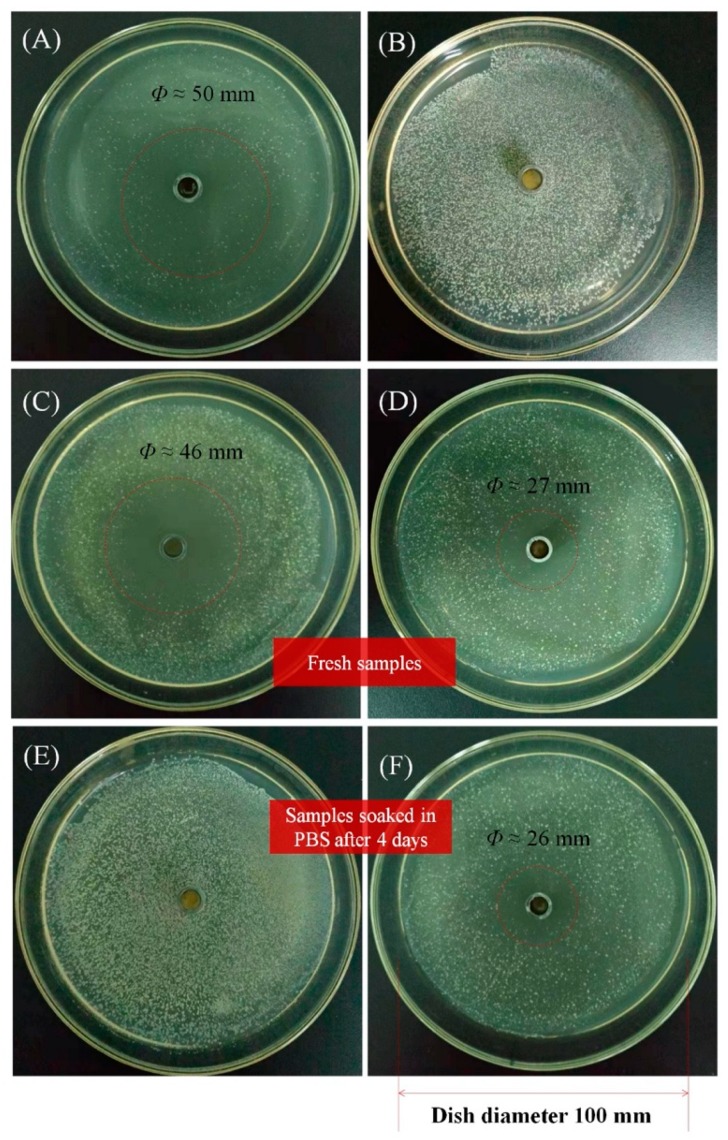
Antibacterial properties of different samples against *S. aureus*. (**A**) CEO solution (2 mg/mL) in ethanol, (**B**) zein film containing blank MCM-41, (**C**) fresh CEO/zein film, (**D**) fresh film nanocomposite, (**E**) CEO/zein film soaked in neutral PBS solution for four days and a (**F**) film nanocomposite soaked in neutral PBS solution for four days.

Figure 6 shows the antibacterial effects of a CEO/zein film (1 *w*/*w*% CEO) and CEO film nanocomposite (1 *w*/*w*% CEO@MCM-41) against *S. aureus*, as well as a CEO solution (2 mg/mL in ethanol) and blank film nanocomposite (1 *w*/*w*% MCM-41 nanoparticles) for the control. The large inhibition zone in Figure 6A demonstrated the significant antibacterial effect of CEO against *S. aureus*, while the blank film nanocomposite had no such effect according to Figure 6B. Shown in Figure 6C,D, it can be seen that the fresh CEO/zein film had a stronger antibacterial effect than that of the fresh CEO film nanocomposite, attributing to more CEO being released in the same period from the CEO/zein film. However, after being soaked in PBS solution for 4 days, the CEO/zein film lost its antibacterial effect (Figure 6E), while the film nanocomposite remained with a similar antibacterial effect as in the beginning (Figure 6F). The sustained but much slower release of CEO from the film nanocomposite was the crucial factor for this long-term antibacterial effect, which is very important for a medicine/food package.

## 4. Conclusions

A long-term antibacterial film nanocomposite containing CEO for medicine or food packaging applications was prepared in this study. The method of modified supercritical CO_2_ impregnation was used to load CEO into MCM-41 silica nanoparticles, and the drug load of CEO@MCM-41 was up to 44.5 ± 0.6 *wt*%. According to the SEM observation, MCM-41 particles around 50 nm were dispersed uniformly in the polyporous matrix of the zein film. The TS of the zein film was reinforced with the addition of MCM-41 particles, while the EAB% decreased sharply when the content of MCM-41 was higher than 1.0 *wt*%. The release of CEO from the film nanocomposite was controlled under all extreme test conditions. Whatever was under high humidity (80%) or continuous illumination conditions in air, the cumulative release amounts of CEO were below 5% after 10 days, while CEO only released 5.5% after 24 h in PBS solution. After being soaked in PBS solution for 4 days, the CEO film nanocomposite still had a strong antibacterial effect against *S. aureus*, the same as the fresh one. It can be concluded that the CEO film nanocomposite can be proposed as a promising long-term antibacterial natural packaging material for medicine and food.

## Figures and Tables

**Table 1:** Results of BET and BJH determination for MCM-41 and CEO@ MCM-41.

Name	MCM-41	CEO@MCM-41
BET surface area	596.36 m^2^/g	264.47 m^2^/g
BJH adsorption surface area	857.53 m^2^/g	407.73 m^2^/g
BJH desorption surface area	868.25 m^2^/g	455.03 m^2^/g
BJH adsorption pore volume	1.56 cm^3^/g	0.46 cm^3^/g
BJH desorption pore volume	1.56 cm^3^/g	0.82 cm^3^/g
BJH adsorption pore width	7.28 nm	4.54 nm
BJH desorption pore width	7.19 nm	7.19 nm

**Table 2:** Film thickness of the zein-based film.

Samples	MCM-41 Contents (%)	Thickness (μm)
Zein film (blank)	0	186.0 ± 16.2
CEO/zein film	-	183.3 ± 16.4
Film nanocomposite	0.5%	218.1 ± 21.6
Film nanocomposite	1.0%	216.5 ± 16.7
Film nanocomposite	2.0%	223.5 ± 11.1
Film nanocomposite	3.0%	227.2 ± 15.2
Film nanocomposite	4.0%	235.6 ± 23.9
Film nanocomposite	5.0%	243.1 ± 19.8

## Supplementary Materials

The following are available online at https://www.mdpi.com/1999-4923/12/2/169/s1, Figure S1: HPLC profile of CEO.

## References

[1] Garavand F., Rouhi M., Razavi S.H., Cacciotti I., Mohammadi R. (2017). Improving the integrity of natural biopolymer films used in food packaging by crosslinking approach: A review. Int. J. Biol. Macromol..

[2] Wang H., Qian J., Ding F. (2018). Emerging Chitosan-Based Films for Food Packaging Applications. J. Agric. Food Chem..

[3] Youssef A., El-Sayed S. (2018). Bionanocomposites materials for food packaging applications: Concepts and future outlook. Carbohydr. Polym..

[4] Muller J., González-Martínez C., Chiralt A. (2017). Combination of poly(lactic) acid and starch for biodegradable food packaging. Materials.

[5] Atarés L., Chiralt A. (2016). Essential oils as additives in biodegradable films and coatings for active food packaging. Trends Food Sci. Technol..

[6] Ribeiro-Santos R., Andrade M., Melo N.R.D., Sanches-Silva A. (2017). Use of essential oils in active food packaging: Recent advances and future trends. Trends Food Sci. Technol..

[7] Acevedo-Fani A., Salvia-Trujillo L., Rojas-Graü M.A., Martín-Belloso O. (2015). Edible films from essential-oil-loaded nanoemulsions: Physicochemical characterization and antimicrobial properties. Food Hydrocoll..

[8] Yuan G., Chen X., Li D. (2016). Chitosan films and coatings containing essential oils: The antioxidant and antimicrobial activity, and application in food systems. Food Res. Int..

[9] Hafsa J., Smach M.A., Ben Khedher M.R., Charfeddine B., Limem K., Majdoub H., Rouatbi S. (2016). Physical, antioxidant and antimicrobial properties of chitosan films containing Eucalyptus globulus essential oil. LWT Food Sci. Technol..

[10] Khaneghah A.M., Hashemi S.M.B., Limbo S. (2018). Antimicrobial agents and packaging systems in antimicrobial active food packaging: An overview of approaches and interactions. Food Bioprod. Process..

[11] Cadena M.B., Preston G.M., Van der Hoorn R.A.L., Flanagan N.A., Townley H.E., Thompson I.P. (2018). Enhancing cinnamon essential oil activity by nanoparticle encapsulation to control seed pathogens. Ind. Crops Prod..

[12] Liu F., Avena-Bustillos R.J., Chiou B.S., Li Y., Ma Y., Williams T.G., Wood D.F., McHugh T.H., Zhong F. (2017). Controlled-release of tea polyphenol from gelatin films incorporated with different ratios of free/nanoencapsulated tea polyphenols into fatty food simulants. Food Hydrocoll..

[13] Ghaderi-Ghahfarokhi M., Barzegar M., Sahari M.A., Ahmadi Gavlighi H., Gardini F. (2017). Chitosan-cinnamon essential oil nano-formulation: Application as a novel additive for controlled release and shelf life extension of beef patties. Int. J. Biol. Macromol..

[14] Bernardos A., Marina T., Žáček P., Pérez-Esteve E., Martínez-Mañez R., Lhotka M., Kouřimská L., Pulkrábek J., Klouček P. (2015). Antifungal effect of essential oil components against Aspergillus niger when loaded into silica mesoporous supports. J. Sci. Food Agric..

[15] Chan A.C., Cadena M.B., Townley H.E., Fricker M.D., Thompson I.P. (2017). Effective delivery of volatile biocides employing mesoporous silicates for treating biofilms. J. R. Soc. Interface.

[16] Vahedikia N., Garavand F., Tajeddin B., Cacciotti I., Jafari S.M., Omidi T., Zahedi Z. (2019). Biodegradable zein film composites reinforced with chitosan nanoparticles and cinnamon essential oil: Physical, mechanical, structural and antimicrobial attributes. Colloids Surf. B Biointerfaces.

[17] Champeau M., Thomassin J.M., Tassaing T., Jérôme C. (2015). Drug loading of polymer implants by supercritical CO_2_ assisted impregnation: A review. J. Control. Release.

[18] Sovova H., Sajfrtova M., Topiar M. (2017). Supercritical CO_2_ extraction of volatile thymoquinone from Monarda didyma and *M. fistulosa* herbs. J. Supercrit. Fluid.

[19] Da Silva R.P.F.F., Rocha-Santos T.A.P., Duarte A.C. (2016). Supercritical fluid extraction of bioactive compounds. Trac Trends Anal. Chem..

[20] Jia J., Liu X., Wu K., Zhou X., Ge F. (2019). Loading zedoary oil into pH-sensitive chitosan grafted mesoporous silica nanoparticles via gate-penetration by supercritical CO_2_ (GPS). J. CO2 Util..

[21] Ahmed J., Mulla M.Z., Arfat Y.A. (2016). Thermo-mechanical, structural characterization and antibacterial performance of solvent casted polylactide/cinnamon oil composite films. Food Control..

[22] Xu T., Gao C., Yang Y., Shen X., Huang M., Liu S., Tang X. (2018). Retention and release properties of cinnamon essential oil in antimicrobial films based on chitosan and gum Arabic. Food Hydrocoll..

[23] Niedermayer S., Weiss V., Herrmann A., Schmidt A., Datz S., Müller K., Wagner E., Bein T., Bräuchle C. (2015). Multifunctional polymer-capped mesoporous silica nanoparticles for pH-responsive targeted drug delivery. Nanoscale.

[24] Noshirvani N., Ghanbarzadeh B., Gardrat C., Rezaei M.R., Hashemi M., Coz C.L., Coma V. (2017). Cinnamon and ginger essential oils to improve antifungal, physical and mechanical properties of chitosan-carboxymethyl cellulose films. Food Hydrocoll..

[25] Atef M., Rezaei M., Behrooz R. (2015). Characterization of physical, mechanical, and antibacterial properties of agar-cellulose bionanocomposite films incorporated with savory essential oil. Food Hydrocoll..

