# Supplementary material for: Zein/MCM-41 Nanocomposite Film Incorporated with Cinnamon Essential Oil Loaded by Modified Supercritical CO2 Impregnation for Long-Term Antibacterial Packaging
Source: Pharmaceutics. 2020 Feb 18;12(2):169. doi: 10.3390/pharmaceutics12020169 (PMC7076511; doi:10.3390/pharmaceutics12020169)
Supplement: Supplementary file 1 [file pharmaceutics-12-00169-s001.pdf]

pharmaceutics

# Supplementary Materials: Zein /MCM-41 Nanocomposite Film Incorporated with Cinnamon Essential Oil Loaded by Modified Supercritical $CO_2$ Impregnation for Long-Term Antibacterial Packaging

**Xiaojing Liu, Jingfu Jia, Shulei Duan, Xue Zhou, Anya Xiang, Ziling Lian, and Fahuan Ge**

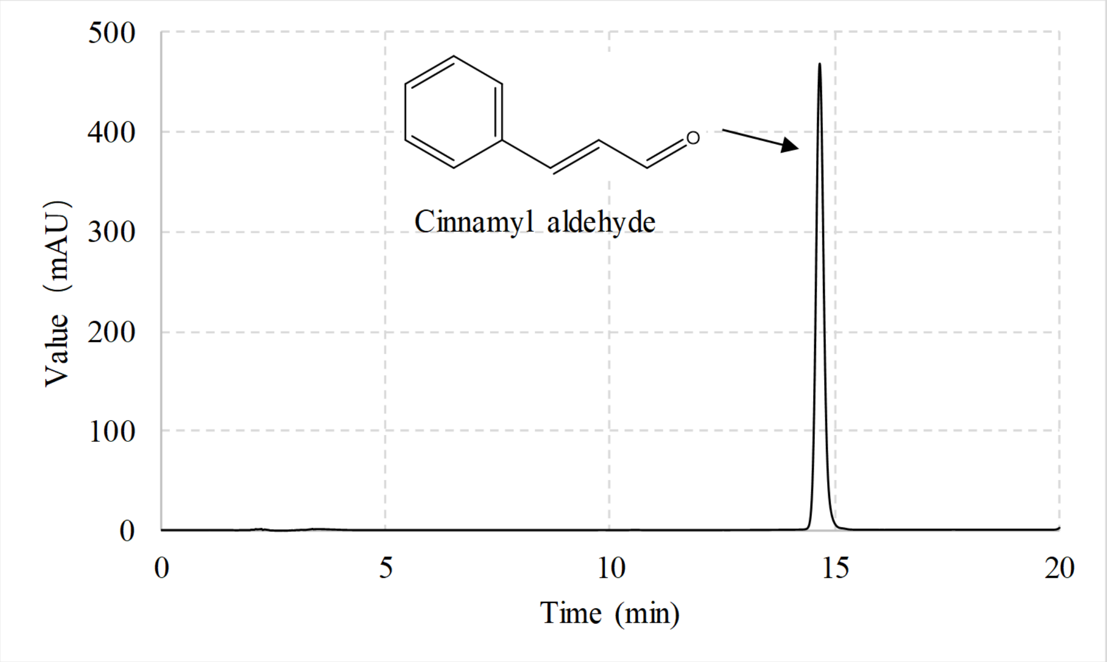


**Figure S1.** HPLC profile of CEO.
